# Lifestyle and metabolic risk factors, and diabetes mellitus prevalence in European countries from three waves of the European Health Interview Survey

**DOI:** 10.1038/s41598-024-62122-y

**Published:** 2024-05-21

**Authors:** Nóra Kovács, Balqees Shahin, Carlos Alexandre Soares Andrade, Nour Mahrouseh, Orsolya Varga

**Affiliations:** 1 Department of Public Health and Epidemiology, Faculty of Medicine, University of Debrecen, Debrecen, Hungary; 2 Doctoral School of Health Sciences, University of Debrecen, Debrecen, Hungary; 3 Syreon Research Institute, Budapest, Hungary

**Keywords:** Risk factors, Epidemiology

## Abstract

Population shift towards healthier lifestyles can help reduce the burden of type 2 diabetes mellitus (DM), therefore understanding and monitoring the lifestyle-related risk factors are crucial for setting up effective preventive strategies and disease management. The present study aimed to explore the changes in prevalence of DM and major risk factors including smoking, physical activity, fruit and vegetable consumption, as well as body mass index (BMI) over three waves of European Health Interview Survey, and to investigate the association between risk factors and presence of DM across 11 European Union member states. Poisson regression models were used to evaluate the association between risk factors and DM, adjusted for demographic and socioeconomic variables. The estimated age-standardized prevalence of DM increased from 7.01% in 2009 to 7.96% in 2019, with substantial increase in subgroups with higher BMI and unhealthy lifestyle including physically inactive people, or current smokers. Obesity and overweight and physical inactivity were significantly associated with DM in all survey waves. Our findings underline that obesity prevention and weight loss promotion along with physical activity promotion are the subject of lifestyle interventions to reduce the burden of DM in EU member states.

## Introduction

Diabetes mellitus (DM) represents a major public health challenge in both developed and developing countries^1^. In Europe, an estimated number of 61 million people aged 20–79 years have DM, and this number is projected to increase to 69 million by 2045 (from 9.2 to 10.4%). In 2021, approximately 1.1 million death was attributed to DM or its complications among people aged 20–79 years^2^. The economic burden of DM is also substantial, with profound implications for healthcare systems and society^3^. According to a report by the International Diabetes Federation, the estimated total cost of DM in Europe reached USD 189 billion in 2021^2^.

DM covers a wide range of metabolic disorders, the most common classifications distinguish Type 1 DM, Type 2 DM, and gestational diabetes.^4^ Type 2 DM accounts for 90% of all cases of DM^2^ which is largely preventable due to its modifiable risk factors including diet, physical activity (PA), alcohol consumption and smoking, body mass index (BMI)^5,6^. Since strong evidence indicate that healthy lifestyle can reduce the disease burden due to type 2 DM^5,7^, understanding and monitoring the lifestyle-related risk factors are crucial for setting up effective preventive strategies and management.

The European Union (EU) has a long history in the fight against DM. Although healthcare services are regulated by the member states and the EU cannot directly legislate on health, it can act effectively, for example in the field of DM prevention^8^. In its strategies, the EU generally does not distinguish between types of DM, regardless of their aetiology. As one of the first DM measures, the St Vincent Declaration, published in 1989 as a result of an international conference, set targets and 5-year objectives to improve the quality of life and life expectancy of people with DM and to reduce the serious complications associated with the disease^9^. The current (2021) framework document "Blueprint for Action on Diabetes in the European Union" sets out policy options and recommendations, outlining what member states and the EU should do. It outlines the societal and legislative changes that are essential, e.g. to reduce the risk of developing type 2 DM and diabetes-related complications, and those needed to ensure appropriate measures and support for healthy lifestyle choices and equal access to care for all^10^.

Having sufficient data for evidence-based policy development is crucial since it makes possible to decrease the uncertainty about the course of action in policy design. Despite the recognition of high burden of DM and its associated lifestyle risk factors across the member states, limited data challenge effective policy development at EU level. Available studies have highlighted regional and national variations in both the prevalence and trends of DM^3,11,12^ and that the weight of individual risk factors differs between EU countries.

The data scarcity is partly remedied by the European Health Interview Survey (EHIS), which is carried out every five years and allows for comparative assessment at least at intervals. EHIS focuses on critical aspects of population health and provide European-wide comparable data to assess the prevalence of DM and the associated risk factors^13^. The utilisation of large population-based representative samples and standardised data collection allows detailed comparisons of lifestyle and metabolic risk factors across diverse European populations, which are often not available in routine settings. This approach is essential to tackle the prevalence of diseases like DM and its risk factors. It helps to create informed policies and targeted interventions.

The aim of the present study was to explore the changes in DM prevalence and major risk factors associated with lifestyle including smoking, PA, and fruit and vegetable consumption, as well as BMI over three waves of EHIS. Additionally, we aimed to investigate the association between risk factors and presence of DM across 11 EU member states.

## Methods

### Study design and data source

A repeated cross-sectional study was carried out using data derived from three waves of EHIS 1, 2 and 3^13^. The first EHIS was carried out between 2006 and 2009 in 17 EU member states. The second wave of EHIS was conducted in all 28 EU member states together with Iceland, Norway, and Turkey between 2013 and 2015. The third wave of survey (EHIS 3) was collected in 2019 in the same countries as in the EHIS 2 along with Albania and Serbia. The EHIS is a population-based survey, which provides data on health status, healthcare use and health determinants across European countries. The EHIS targets individuals aged 15 years and older living in private households. Each country selected a nationally representative sample from population registers, censuses, dwelling registers or other sources. The most common sampling design was a multi-stage stratified or systematic (cluster) sampling design with the individual being the ultimate sampling unit. Weighting factors were calculated by each EU member state to reduce non-response bias, to reflect the sample design and to ensure that the sample accurately reflected the structure of the population^14^. Detailed information on the methodology is available elsewhere^14,15^. The microdata used for the analyses were provided by the Statistical Office of the European Union (Eurostat).

### Study population

Participants with missing data for any of the studied variables were excluded from the analyses. A total of n = 22,970 (21.4%) participants were excluded for 2009, n = 7,436 (6.8%) for 2014, and n = 10,453 (9.5%) for 2019. The final samples included in our analyses consisted of N = 84,239 (EHIS 1), N = 101,355 (EHIS 2) and N = 99,006 (EHIS 3) participants in 2009, 2014 and 2019, respectively. The study population included adults aged 20 years or older from 11 EU Member States: Bulgaria, Cyprus, Czech Republic, Greece, Spain, Hungary, Latvia, Poland, Romania, Slovenia, and Slovakia. Only countries with data available for each wave of survey were included in this study. Responses from Austria, Belgium and Estonia were not taken into account as data on some risk factors were missing. Malta was also not included due to unavailability of weights in EHIS 1 wave.

### Study variables

All data of the respondents were self-reported. Self-reported DM was used as outcome variable. Participants were asked “During the past 12 months, have you had diabetes?”. Respondents answering with “yes” were considered as having DM.

Demographic characteristics were sex and age (20 to 44, 45 to 64, and 65 and above). Socioeconomic characteristics included education level, degree of urbanization, and labour status. Individuals’ education level was assessed according to the International Standard Classification of Education (ISCED)^16,17^. In each survey, we collapsed the levels of education to three main levels: ISCED levels 0 to 2 (0 to 3 in EHIS 1) were merged into a primary or less than primary education group, ISCED levels 3 to 4 (4 to 5 in EHIS 1) into a secondary education group, and levels 5 to 8 (6 to 7 in EHIS 1) into a higher education group. Degree of urbanization were divided into two categories: urban area included densely populated area and intermediate-populated area (in EHIS 1 and 2), and cities and towns and suburbs (in EHIS 3); rural areas included thinly populated area (in EHIS 1 and 2) and rural areas (in EHIS 3). Labour status was classified as employed, unemployed and other (including student, pupil, retired, fulfilling domestic tasks, permanently disabled, compulsory military, community service, and other).

Five risk factors were assessed in the present study according to self-reported answers: BMI, and lifestyle risk factors including smoking status, PA and consumption of fruit and vegetable. BMI was calculated as body weight in kilogrammes divided by height in metres squared (kg/m^2^). In the analysis, participants were divided into two groups based on BMI: underweight or normal (BMI < 25kg/m^2^), and overweight or obese (BMI ≥ 25 kg/m^2^). Smoking status was classified as non-smoker and current smoker, including daily or occasional smokers. Regarding physical activity, time devoted to moderate level physical activity per week (without walking) and frequency of walking at least 10 min per day were calculated separately. In EHIS 1, time spent on moderate level physical activity per week was considered. In EHIS 2 and 3, the duration of aerobic level physical activity was determined using the EHIS-Physical Activity Questionnaire (EHIS-PAQ)^18,19^. Health-enhancing physical activity (HEPA) was computed by summing up the minutes per week spent on sports, fitness or recreational (leisure) physical activities and cycling^18^. In accordance with the guidelines^20,21^, PA indicator was dichotomized as performing at least 150 min moderate-intensity physical activity per week or not. Frequency of walking at least 10 min per day was categorized as never, one to six days per week, and every day. Frequency of eating fruits and vegetables per week were used separately in the analyses. Fruit and vegetable intake were categorized as once or more a day, one to six times a week and less than once a week or never.

### Statistical analysis

Descriptive statistics were used to describe the characteristics of the sample populations for each survey. We calculated unweighted absolute number and weighted proportions of participants for each survey using sampling weights available in the database of EHIS surveys. Differences between groups were tested using chi-square tests.

The crude prevalence of DM was estimated. To account for differences in the age structure of populations and to allow adequate comparison of prevalence estimates, direct standardization was applied using the 2013 revision of the European Standard Population. Age-standardized prevalence of diabetes for each risk factor category was calculated for the population aged 20 years and older by using 5 year age groups.^22^.

Poisson regression models were used to evaluate the association between risk factors and DM, adjusted for demographic and socioeconomic variables. Prevalence ratios (PRs) with 95% confidence intervals (CIs) were presented. We used the three datasets separately for analyses, as well as a pooled dataset was used adding an interaction term between survey year and risk factors to the model to test whether the prevalence of DM by risk factor variables differed over time. Furthermore, to investigate differences in risk factor prevalence between European regions over time, the 11 countries were categorized into the following two groups: Central and Eastern European countries (Bulgaria, Czech Republic, Hungary, Latvia, Poland, Romania, Slovenia, Slovakia) and Southern countries (Cyprus, Greece, Spain), according to EuroVoc classification. Such geographical division reveals important socioeconomic differences between these two European regions^23^.

Sampling weights were applied using the “svy” command in Stata. Akaike Information Criteria (AIC) and Bayesian Information Criteria (BIC) were used to assess model performance. A p-value less than 0.05 was considered statistically significant. Statistical analyses were performed by using STATA IC version 13.0 software (StataCorp LP, College Station, Texas, USA). All the figures were created by using Microsoft Excel (Microsoft 365).^24^.

### Ethics statements

This study is based on secondary analysis of a public and anonymised dataset, which had obtained ethics approval on a national level by the institutions responsible for the survey implementation and therefore required no additional ethics approval. The study was carried out in accordance with relevant guidelines and regulations. The EHIS was carried out according to the Regulation (EC) No. 1338/2008 of the European Parliament and of the Council of 16th December 2008. The data collected in EHIS wave 2 by each country have been conducted according to the Commission Regulation (EU) No. 141/2013. EHIS wave 3 was conducted according Commission Regulation (EU) No. 2018/255. Participants gave informed consent to participate in the study before taking part. Our study is based on approved Eurostat research proposal project RPP 266/2020-LFS-EHIS.

## Results

### Characteristics of the study population

Table 1 shows the number of participants and weighted percentages by presence of DM according to demographic and socioeconomic variables and risk factors.

**Table 1:** Characteristics of study population by diabetes status and survey.

		EHIS 1					EHIS 2					EHIS 3				
		DM (n = 5615)		non-DM (n = 78,624)			DM (n = 8229)		non-DM (n = 93,126)			DM (n = 9,221)		non-DM (n = 89,785)		
		%	n	%	n	*p*-value*	%	n	%	n	*p*-value*	%	n	%	n	*p*-value*
Sex	Male	46.5	2437	47.8	35,227	**0.041**	47.5	3630	47.6	41,726	0.225	47.4	4159	47.5	40,611	0.814
	Female	53.5	3178	52.3	43,397		52.5	4599	52.4	51,400		52.6	5062	52.6	49,174	
Age	20–44	7.3	300	50.1	33,899	** < 0.001**	6.9	397	47.1	36,833	** < 0.001**	5.6	329	44.0	31,350	** < 0.001**
	45–64	40.9	2171	33.6	28,581		36.3	2678	34.3	33,562		34.2	2718	35.6	32,878	
	65 +	51.8	3144	16.3	16,144		56.8	5154	18.6	22,731		60.2	6174	20.4	25,557	
Education level	Primary	53.9	3113	30.9	24,995	** < 0.001**	49.8	4074	28.9	26,644	** < 0.001**	42.3	3806	25.0	22,343	** < 0.001**
	Secondary	36.4	1976	47.8	38,148		38.8	3264	46.6	44,952		43.3	4043	46.9	42,494	
	Tertiary	9.7	526	21.4	15,481		11.4	891	24.5	21,530		14.4	1372	28.1	24,948	
Residential area	Rural	40.8	2613	39.6	36,704	0.831	34.4	3254	34.4	35,741	**0.038**	29.6	3317	29.5	31,056	**0.008**
	Urban	59.2	3002	60.4	41,920		65.6	4975	65.7	57,385		70.4	5904	70.5	58,729	
Labour status	Employed	19.9	979	56.1	41,654	** < 0.001**	18.7	1299	53.5	46,260	** < 0.001**	21.2	1549	57.2	45,846	** < 0.001**
	Other	76.3	4473	35.2	31,206		76.0	6615	36.4	38,767		75.5	7411	36.0	38,726	
	Unemployed	3.9	163	8.8	5764		5.2	315	10.1	8099		3.3	261	6.7	5213	
Body mass index	< 25kg/m^2^	22.2	1219	46.6	35,815	** < 0.001**	21.6	1760	46.3	41,398	** < 0.001**	20.3	1839	44.1	37,759	** < 0.001**
	≥ 25 kg/m^2^	77.8	4396	53.4	42,809		78.4	6469	53.7	51,728		79.7	7382	55.9	52,026	
Frequency of eating vegetables	Once or more a day	65.1	3689	61.2	48,797	** < 0.001**	51.2	4221	46.5	43,307	** < 0.001**	48.7	4287	44.1	38,509	** < 0.001**
	1–6 times a week	29.8	1665	34.6	26,666		44.0	3639	49.0	45,636		47.4	4535	52.4	47,677	
	Less than once a week or never	5.1	261	4.3	3161		4.8	369	4.5	4183		3.9	399	3.6	3599	
Frequency of eating fruits	Once or more a day	71.9	3931	63.6	49,528	** < 0.001**	62.8	4873	54.4	48,697	** < 0.001**	60.0	5204	52.5	44,916	** < 0.001**
	1–6 times a week	21.4	1288	29.1	23,424		31.0	2827	39.2	38,134		33.0	3344	40.8	38,702	
	Less than once a week or never	6.8	396	7.3	5672		6.3	529	6.4	6295		7.0	673	6.7	6167	
Physical activity#	≥ 150 min moderate PA per week	41.7	2358	56.3	44,808	** < 0.001**	13.5	1012	26.7	22,743	** < 0.001**	14.9	1236	28.5	22,172	** < 0.001**
	< 150 min moderate PA per week	58.3	3257	43.7	33,816		86.5	7217	73.3	70,383		85.1	7985	71.5	67,613	
Frequency of walking for 10 min per week	Never	22.9	1440	15.5	13,848	** < 0.001**	23.9	2142	13.8	14,882	** < 0.001**	20.2	2324	11.3	14,375	** < 0.001**
	Everyday	48.0	2563	50.8	38,450		38.8	2999	47.0	42,008		40.0	3439	48.0	40,644	
	1–6 days a week	29.2	1612	33.7	26,326		37.4	3088	39.3	36,236		39.8	3458	40.8	34,766	
Smoking status	Non-smoker	81.8	4680	67.1	54,098	** < 0.001**	82.0	6906	71.2	67,468	** < 0.001**	81.5	7646	73.1	66,204	** < 0.001**
	Current smoker	18.2	935	32.9	24,526		18.1	1323	28.8	25,658		18.5	1575	26.9	23,581	

%: weighted proportions; n: unweighted number of participants; DM: diabetes mellitus; EHIS: European Health Interview Survey; PA: physical activity.

*Significant differences are shown is bold.

^#^Physical activity was calculated in EHIS 1 according to time spent on moderate level physical activity per week; in EHIS 2 and EHIS 3, health-enhancing physical activity (HEPA) was computed by summing up the minutes per week spent on sports, fitness or recreational (leisure) physical activities and cycling.

In 2009, 2014, and 2019, there was a significant difference in proportion of participants across age groups. The majority of respondents with DM in each year were aged 65 and over, accounting for 51.8%, 56.8%, and 60.2%, respectively. Among DM adults, the percentage of respondents with primary education was significantly higher, however, this percentage declined over the study period. Over the three waves, the proportion of participants with DM living in urban areas increased from 59.2 to 70.4%. Regarding labour status, higher proportion of participants with DM reported being in the “Other" category—which included retired individuals, people with disabilities, etc. – compared to those participants who did not report DM (76.3% in 2009, 76% in 2014 and 75.5% in 2019).

Across the three waves of the survey, the majority of participants who reported living with DM (77.8%, 78.4% and 79.7% respectively) also reported a higher BMI than those who did not report living with DM. Respondents with DM reported a significantly higher frequency of eating fruits (71.9%, 62.8% and 60.0%, respectively) or vegetables (65.1%, 51.2% and 48.7%, respectively) once or more a day compared to those without DM. Walking for 10 min every day was reported by around half of respondents, with a slightly higher frequency among non-DM individuals. The proportion of those who engaged at least 150 min of moderate physical activity per week was generally low at each wave of the survey, and a lower proportion was reported by participants with DM compared with the non-DM population. Current smoking was less common among people living with DM (18.2%, 18.1% and 18.5%, respectively), than for non-DM individuals (Table 1).

### Age-standardised prevalence of diabetes mellitus by risk factors

In the study population, the crude prevalence of DM was 6.7%, 8.1%, and 9.3% in 2009, 2014 and 2019, respectively. The overall estimated age-standardized prevalence of DM increased from 7.01% in 2009 to 7.96% in 2019 in the population aged 20 years and older (Table 2).

**Table 2:** Age-standardised prevalence of diabetes mellitus by socioeconomic variables and risk factors by waves of EHIS.

		EHIS1		EHIS2		EHIS3	
		%	95% CI	%	95% CI	%	95% CI
DM	Yes	7.01	(7.01–7.02)	7.70	(7.7–7.71)	7.97	(7.96–7.97)
Sex	Male	7.22	(7.21–7.23)	8.16	(8.16–8.17)	8.48	(8.47–8.49)
	Female	6.79	(6.78–6.8)	7.29	(7.28–7.3)	7.52	(7.52–7.53)
Education level	Primary	7.84	(7.83–7.85)	8.69	(8.68–8.7)	8.91	(8.9–8.92)
	Secondary	6.79	(6.78–6.8)	7.56	(7.56–7.57)	8.00	(7.99–8.01)
	Tertiary	5.08	(5.07–5.1)	5.95	(5.94–5.97)	6.35	(6.34–6.36)
Labour status	Employed	3.43	(3.41–3.45)	4.55	(4.53–4.58)	5.35	(5.31–5.39)
	Other	8.42	(8.41–8.43)	9.38	(9.37–9.39)	9.50	(9.49–9.51)
	Unemployed	9.02	(8.78–9.26)	5.80	(5.76–5.85)	6.95	(6.8–7.1)
Residential area	Rural	6.89	(6.88–6.9)	7.40	(7.39–7.41)	7.64	(7.63–7.65)
	Urban	7.12	(7.11–7.12)	7.87	(7.87–7.88)	8.12	(8.11–8.12)
Body mass index	< 25 kg/m^2^	4.61	(4.6–4.62)	4.92	(4.92–4.93)	5.07	(5.06–5.08)
	≥ 25 kg/m^2^	8.13	(8.12–8.14)	9.13	(9.13–9.14)	9.39	(9.38–9.4)
Frequency of eating vegetables	Once or more a day	7.16	(7.15–7.16)	8.01	(8–8.01)	8.50	(8.49–8.5)
	1–6 times a week	6.56	(6.55–6.57)	7.36	(7.35–7.37)	7.48	(7.48–7.49)
	Less than once a week or never	8.51	(8.48–8.54)	8.04	(8.02–8.07)	8.22	(8.2–8.25)
Frequency of eating fruits	Once or more a day	7.23	(7.22–7.23)	8.08	(8.07–8.09)	8.34	(8.34–8.35)
	1–6 times a week	6.33	(6.32–6.34)	7.02	(7.01–7.03)	7.21	(7.2–7.22)
	Less than once a week or never	7.23	(7.21–7.26)	7.94	(7.91–7.96)	9.05	(9.02–9.07)
Physical activity	≥ 150 min moderate PA per week	5.92	(5.91–5.93)	6.05	(6.04–6.06)	6.27	(6.26–6.29)
	< 150 min moderate PA per week	8.00	(7.99–8.01)	8.06	(8.05–8.07)	8.40	(8.39–8.41)
Frequency of walking for at least 10 min continuously per week	Never	8.70	(8.69–8.72)	10.13	(10.12–10.15)	10.78	(10.76–10.8)
	Everyday	6.62	(6.61–6.63)	6.75	(6.74–6.75)	7.18	(7.18–7.19)
	1–6 days a week	6.49	(6.47–6.5)	7.56	(7.56–7.57)	7.89	(7.89–7.9)
Smoking status	Non-smoker	7.27	(7.27–7.28)	7.81	(7.8–7.81)	8.03	(8.02–8.04)
	Current smoker	6.11	(6.09–6.12)	7.05	(7.04–7.07)	7.63	(7.62–7.65)

CI: Confidence interval; DM: diabetes mellitus; EHIS: European Health Interview Survey; PA: physical activity.

The age-standardised prevalence of DM was almost twice as high in people with a BMI ≥ 25 kg/m^2^ as in those with a BMI < 25 kg/m^2^, and increased from 8.13 to 9.39% over the waves. Similarly, the age-standardized prevalence of DM was higher among those who consumed fruits once or more a day in 2009 and 2014, and vegetables once or more a day in 2014 and 2019 compared with non-daily consumers. In general, a lower age-standardized prevalence of DM was observed among participants who performed at least 150 min of moderate-intensity physical activity per week and 10 min of walking every day, compared to those who performed less physical activity per week. The prevalence of DM was higher among non-smokers than among current smokers, however, the difference in prevalence between smokers and non-smokers decreased over survey waves (Table 2). The Supplementary material (see Supplementary Fig. S1-S3) contains the estimated age-standardized prevalence of DM by risk factors for each country and survey wave.

### Association between risk factors and diabetes mellitus prevalence over time

The results of Poisson regression analysis are shown in Table 3. DM was significantly associated with being overweight or obese (BMI ≥ 25 kg/m^2^) (2009: PR 1.75; 95% CI 1.61–1.89; 2014: PR 1.83; 95% CI 1.72–1.95; 2019: PR 1.85; 95% CI 1.73–1.98), and with performing less than 150 min of moderate physical activity per week (2009: PR 1.24; 95% CI 1.16–1.34; 2014: PR 1.39; 95% CI 1.27–1.51; 2019: PR 1.4; 95% CI 1.29–1.52) across the three survey waves. Current smoking (2009: PR 0.79; 95% CI 0.71–0.86; 2014: PR 0.9; 95% CI 0.83–0.97; 2019: PR 0.93; 95% CI 0.86–0.99) and walking for 10 min every day (2009: PR 0.87; 95% CI 0.79–0.94; 2014: PR 0.73; 95% CI 0.68–0.78; 2019: PR 0.71; 95% CI 0.66–0.76) or one to six days per week (2009: PR 0.87; 95% CI 0.79–0.95; 2014: PR 0.82; 95% CI 0.76–0.87; 2019: PR 0.78; 95% CI 0.73–0.84) was negatively associated with having DM in each wave of the survey. Individuals who ate fruits one to six times a week were less likely to report DM in 2014 (PR 0.87; 95% CI 0.81–0.93) and 2019 (PR 0.88; 95% CI 0.82–0.94) than those who ate fruits daily. Individuals who consumed vegetables one to six times per week were also less likely to report DM in 2009 (PR 0.92; 95% CI 0.85–0.99) and 2019 (PR 0.9; 95% CI 0.84–0.95) compared with daily consumers (Table 3).

**Table 3:** Association between risk factors and diabetes mellitus by waves of EHIS.

		EHIS1		EHIS2		EHIS3		p-value for interaction*
		PR	95% CI	PR	95% CI	PR	95% CI	
Body mass index	< 25kg/m^2^ (reference)							
	≥ 25 kg/m^2^	**1.75**	**(1.61–1.89)**	**1.83**	**(1.72–1.95)**	**1.85**	**(1.73–1.98)**	0.410
Frequency of eating vegetables	Once or more a day (reference)							
	1–6 times a week	**0.92**	**(0.85–0.99)**	0.96	(0.89–1.02)	**0.9**	**(0.84–0.95)**	0.363
	Less than once a week or never	1.07	(0.91–1.25)	0.96	(0.83–1.12)	**0.86**	**(0.76–0.99)**	0.146
Frequency of eating fruits	Once or more a day (reference)							
	1–6 times a week	0.93	(0.85–1.02)	**0.87**	**(0.81–0.93)**	**0.88**	**(0.82–0.94)**	0.207
	Less than once a week or never	1.02	(0.89–1.18)	0.96	(0.85–1.08)	**1.12**	**(1.00–1.25)**	0.507
Physical activity#	≥ 150 min moderate PA per week (reference)							
	< 150 min moderate PA per week	**1.24**	**(1.16–1.34)**	**1.39**	**(1.27–1.51)**	**1.4**	**(1.29–1.52)**	**0.008**
Frequency of walking for at least 10 min continuously per week	Never (reference)							
	Everyday	**0.87**	**(0.79–0.94)**	**0.73**	**(0.68–0.78)**	**0.71**	**(0.66–0.76)**	**0.001**
	1–6 days a week	**0.87**	**(0.79–0.95)**	**0.82**	**(0.76–0.87)**	**0.78**	**(0.73–0.84)**	0.076
Smoking status	Non-smoker (reference)							
	Current smoker	**0.79**	**(0.71–0.86)**	**0.9**	**(0.83–0.97)**	**0.93**	**(0.86–0.99)**	**0.044**
ΔAIC			-6,956,911		-9,020,697		-9,392,992	
ΔBIC			-6,956,659		-9,020,440		-9,392,735	

All models were adjusted for sex, age, education level, labour status, residential area, and country.

CI: Confidence interval; Prevalence ratio: PR; PA: physical activity; EHIS: European Health Interview Survey. ΔAIC and ΔBIC indicate the difference between null regression model and full regression model (ΔAIC = AICnull-AICfull, where *AICnull* represents the null model and *AICfull* represents the full model).

Significant results are shown in bold.

*p-value for interaction between survey year and risk factors.

^#^Physical activity was calculated in EHIS 1 according to time spent on moderate level physical activity per week; in EHIS 2 and EHIS 3, health-enhancing physical activity (HEPA) was computed by summing up the minutes per week spent on sports, fitness or recreational (leisure) physical activities and cycling.

In the pooled dataset, a significant interaction of the survey year with moderate level physical activity (*p* = 0.008), walking every day (*p* = 0.001) and smoking (*p* = 0.044) was observed in the multivariable analysis (Table 3).

According to the stratified analysis by region, PA and BMI were found to be associated with DM in all waves, regardless of the region of the country. However, the association between smoking and DM was observed in Central and Eastern European countries, but not in Southern countries. Regarding diet, Southern European countries had a higher consumption of fruits and vegetables compared to Central and Eastern European countries (see Supplementary Table S1).

## Discussion

This is the first study utilizing data from three waves of EHIS to investigate the prevalence of DM with regards to various lifestyle-related risk factors in 11 EU member states between 2009 and 2019. The repeated cross-sectional design allowed the assessment of DM prevalence and its relationship with BMI and lifestyle factors at different time points. We found that a higher proportion of individuals with DM reported healthier behaviour in terms of smoking and fruit and vegetable consumption. However, a higher BMI and less physical activity were also more common among individuals with DM than in people without the disease. The estimated prevalence of DM continued to increase, particularly in subgroups with higher BMI and unhealthy lifestyle including physically inactive people, or current smokers.

The age-standardized prevalence of DM in EU countries showed a progressive increase between 2009 and 2019 (7.01% to 7.97%, respectively). This is line with previous studies which have reported increasing trend in DM prevalence rate over time across EU member states^25–28^. A systematic review including 10 EU member states reported that not only the prevalence of DM is increasing from 2009, but also the incidence and the mortality of the disease^3^. The observed rise in prevalence demonstrates the inefficacy of public health interventions and management of DM^29,30^.

According to our results, the proportion of individuals with DM who completed secondary and tertiary education increased between 2009 and 2019. Despite this increase, individuals with DM still had lower educational levels compared to those without DM. Similar findings have been observed in studies conducted in EU member states, indicating a correlation between lower educational levels and a higher likelihood of DM prevalence^31–33^. Evidence have revealed the crucial role of education in managing and preventing DM^34,35^. Higher educational levels are associated with higher income and improved knowledge of healthy nutrition and behaviour, leading to healthier outcomes, such as satisfactory glycaemic control^36,37^.

Our study reveals a significant disparity in labour status between individuals with and without the disease, however, the labour status did not statistically change between the three waves, indicating that the observed disparities persist over time. Individuals with DM were less likely to have formal jobs compared to individuals without the disease, which can be attributed to the potential impact of DM on functioning and physical health, which may restrict their ability to engage in demanding and structured work^38,39^. These findings emphasize the need for targeted interventions and policies to address the unique challenges faced by individuals with DM in the labour market^40^. The prevalence of DM was slightly higher in urban areas, nonetheless, a higher increase was observed in rural areas between 2009 and 2019. A higher prevalence of DM is reported in urban than in rural areas in most countries around the world^2^, which may be explained by better access to health services resulting in proper diagnosis. Furthermore, an ecological multi-country study suggested a positive link between agglomeration index (as a measure of urbanization) and prevalence of type 2 DM, however, in high income countries the prevalence of type 2 DM was rather related to a more diabetogenic environment comprising obesity and physical inactivity than to urbanization^41^.

Number of studies demonstrated that overweight and obesity are associated with the risk of chronic conditions including type 2 DM^42,43^. High BMI is a known contributor to insulin resistance, which is a key underlying factor in the development of type 2 DM^44^. Our study revealed a substantially increase in the age-standardized prevalence of DM among overweight or obese participants in the study period. Furthermore, the strongest association was observed between DM and elevated BMI (> 25 kg/m^2^) among all investigated modifiable risk factors which underscores the critical need for interventions addressing the complex link between BMI and DM. In most EU member states, more than half of adults are overweight or obese^45^. Existing obesity policies have not been successful, and in EU countries obesity rates are stagnating at best. Failure of traditional obesity control measures indicates the importance of developing a new, non-stigmatic approach to public policy, led by interprofessional teams^46^.

The benefits of regular physical activity on blood glucose control and risk factors for DM complications have shown in previous studies^47,48^. However, lower proportion of patients with DM met the physical activity guidelines in the present study compared to participants without DM, which may suggest that the positive effect of physical activity is less pronounced in the management of DM. Engaging in at least 150 min of moderate-intensity physical activity per week and at least daily 10-min walks were associated with a lower prevalence of DM across all waves. A recent study suggested that there is a wide variation across EU member states regarding physical activity policies, however, majority of countries were without implemented policies^29^.

Participants with DM were less likely to be current smokers than those without DM. However, the prevalence gap between smokers and non-smokers decreased with survey waves. Recent studies also found a lack of the expected positive association between smoking and DM^49,50^. A possible explanation could be that diabetic patients tend to pay more attention to their lifestyle compared to non-diabetic population, suggesting a greater probability to stop smoking in order to follow the physicians’ recommendation^50^. Furthermore, the inverse association was only observed in Central and Eastern European countries which may suggest that the effectiveness of tobacco control measures varies between countries, and also smoking is determined by socioeconomic status^51^. The strong inverse correlation between smoking and DM does not in any way justify the promotion of smoking.

Healthy diet (e.g. high intake of fruit and vegetables) has often been linked to the prevention of non-communicable chronic diseases, including type 2 DM^1^. In the present study, we found a healthier diet (higher fruit- and vegetable intake) among people with DM compared to those without DM. Previous cross-sectional studies also found higher frequency of fruit and vegetable consumption in participants with DM than in non-diabetic population^49,52^, which might be due to greater awareness of healthy eating as a result of doctors’ dietary recommendations during diabetes care management^49^. However, the findings are inconsistent in the literature regarding the association between fruit and vegetable consumption and risk of DM. The results of several meta-analyses including cohort studies indicated that the consumption of fruit and vegetables did not contribute, or only weakly, to a significant reduction in the risk of type 2 DM^53,54^. Furthermore, other studies suggested that the intake of dietary fibre from fruit and vegetables was not associated with the risk of DM^55^. We found that higher intake of fruit and vegetables among patients with DM is more typical of the Southern countries, which points out that significant variations exist within the EU^56^, mainly due to geographical, educational and cultural differences across regions^57–59^.

In summary, the association studies indicate that only high BMI (> = 25 kg/m^2^) and physical inactivity were significantly associated with DM in all survey waves. These findings highlight the importance of preventing obesity, promoting weight loss, and supporting physical activity to reduce the burden of DM in EU member states.The EU member states and the EU itself have a number of policies to promote physical activity and reduce obesity. The Physical Activity Fact Sheet 2021^60^ provides a snapshot of 23 indicators of physical activity and related policy initiatives in EU countries. Educating and promoting physical activity among children and young people is a priority for the EU. Conversely, the European guidelines for improving leisure-time physical activity infrastructure were implemented in only 5 countries^61^. While there are clearly initiatives to promote physical activity, these are not enough. Stronger interventions at EU level to promote physical activity especially in older adults are needed.

The study is based on three comparable surveys over ten-year period. Although this study provides insights into the association between risk factors and DM prevalence in 11 EU member states, it has several limitations. In particular, all data on the variables studied (including health risk behaviours and the presence of DM) are based on self-report measures, which may lead to information and desirability bias, which may have contributed to the unexpected finding of an inverse association between smoking and DM. Respondents may bias self-report of risk factors in a favourable direction^62^, for example, they might answer in a more socially desirable way (saying they are non-smokers even if they are smokers). However, previous studies indicated an agreement between medical records and self-reported DM status^63,64^. As the EHIS does not collect data on type of DM, we could not distinguish between type 1 and type 2 DM in our analysis, although each type has different background pathologies and associated risk factors. However, it is reasonable to assume that type 2 DM is the predominant form in the adult population, and therefore, in our study. Additionally, the cross-sectional study design limits causal inferences, as it is not possible to establish a temporal relationship between risk factors and DM. Since the present study is focusing on specific EU member states, our findings cannot necessarily be generalized to the entire population of the EU. On the other hand, excluding countries and participants with missing data from the analyses may impact our findings.. The included participants may differ from those with missing data, which could lead to an overestimation or underestimation of the prevalence of risk factors in the populations. Additionally, variations in the prevalence of DM and risk factors in the excluded countries may have influenced the results. As per the source^65^, DM is more prevalent in Southern European and Central and Eastern European countries. Therefore, excluding Nordic and Western countries from this study could lead to a higher estimated overall prevalence of the disease. The calculation of guideline recommended physical activity level was not uniform in all waves due to changes in methodology of data collection. In the first wave of EHIS, PA was measured with a modified version of the International Physical Activity Questionnaire – Short Form (IPAQ-SF), collected data on frequency of vigorous-intensity PA, moderate-intensity PA and walking. After revealing difficulties with data collection in wave 1, a short, domain-specific PA questionnaire (EHIS-PAQ) was developed evaluating how far the population is physically active in specific public health relevant settings (work, transport, leisure time)^18^. Therefore, in first wave, we considered moderate-intensity PA, and in second and third wave we calculated health-enhancing PA (HEPA). Such slight difference in survey methodology may affect the interaction calculation.

## Conclusion

This is the first study on DM and metabolic and lifestyle risk factors using the three waves of the EHIS, covering several EU countries. In European countries, individuals with DM commonly exhibit higher BMI and engage in less physical activity in all survey waves. These metabolic and lifestyle factors significantly contribute to the development and management of DM, highlighting the importance of promoting obesity prevention, weight loss, and physical activity to reduce the burden of DM. Studies indicate that lifestyle interventions aimed at weight loss and increased physical activity have been effective in improving glycaemic control and health outcomes for individuals with T2DM^66^. Additionally, creating supportive environments for physical activity, promoting healthy dietary habits, and addressing social inequalities that contribute to unhealthy lifestyles can positively impact the prevention and management of DM at a population level. In the EU, EU-level policies for the primary prevention of DM are necessary alongside national and regional policies, to reverse the unfavourable trend.

### Supplementary Information

Supplementary Information.

## Data Availability

Microdata of EHIS 2009, 2014 and 2019 are available from Eurostat upon request (https://ec.europa.eu/eurostat). The contact person of the database used in our study is Orsolya Varga (varga.orsolya@med.unideb.hu).
