# Supplementary material for: Lifestyle and metabolic risk factors, and diabetes mellitus prevalence in European countries from three waves of the European Health Interview Survey
Source: Sci Rep. 2024 May 21;14:11623. doi: 10.1038/s41598-024-62122-y (PMC11109107; doi:10.1038/s41598-024-62122-y)
Supplement: Supplementary file 1 — Supplementary Information. [file 41598_2024_62122_MOESM1_ESM.docx]

**Supplementary material**

**Supplementary Table S1.** Association between risk factors and diabetes mellitus by waves of EHIS in Central and Eastern European and Southern European countries.

|  |  | EHIS1 | | | |  | EHIS2 | | | |  | EHIS3 | | | |
| --- | --- | --- | --- | --- | --- | --- | --- | --- | --- | --- | --- | --- | --- | --- | --- |
|  |  | CEE | | SOUTHERN | |  | CEE | | SOUTHERN | |  | CEE | | SOUTHERN | |
|  |  | PR | 95% CI | PR | 95% CI |  | PR | 95% CI | PR | 95% CI |  | PR | 95% CI | PR | 95% CI |
| Body mass index | <25kg/m2 (reference) |  |  |  |  |  |  |  |  |  |  |  |  |  |  |
|  | ≥25 kg/m2 | **1.83** | **(1.66-2.03)** | **1.63** | **(1.43-1.84)** |  | **1.92** | **(1.78-2.07)** | **1.68** | **(1.51-1.88)** |  | **2.02** | **(1.87-2.2)** | **1.61** | **(1.43-1.8)** |
| Frequency of eating vegetables | once or more a day (reference) |  |  |  |  |  |  |  |  |  |  |  |  |  |  |
|  | 1-6 times a week | 0.90 | (0.81-1.01) | 0.93 | (0.82-1.05) |  | 1.00 | (0.92-1.08) | **0.88** | **(0.8-0.98)** |  | **0.88** | **(0.82-0.96)** | **0.88** | **(0.8-0.97)** |
|  | less than once a week or never | 1.10 | (0.91-1.34) | 0.97 | (0.75-1.26) |  | 0.93 | (0.79-1.09) | 1.02 | (0.75-1.38) |  | **0.84** | **(0.72-0.97)** | 0.98 | (0.64-1.5) |
| Frequency of eating fruits | once or more a day (reference) |  |  |  |  |  |  |  |  |  |  |  |  |  |  |
|  | 1-6 times a week | 0.98 | (0.88-1.11) | 0.88 | (0.75-1.02) |  | **0.90** | **(0.83-0.98)** | **0.79** | **(0.7-0.89)** |  | 0.93 | (0.85-1.01) | **0.81** | **(0.72-0.92)** |
|  | less than once a week or never | 1.15 | (0.98-1.36) | 0.84 | (0.65-1.09) |  | 1.10 | (0.96-1.26) | **0.72** | **(0.55-0.95)** |  | **1.22** | **(1.07-1.38)** | 0.97 | (0.76-1.23) |
| Physical activity# | >=150 min moderate PA per week (reference) |  |  |  |  |  |  |  |  |  |  |  |  |  |  |
|  | <150 min moderate PA per week | **1.23** | **(1.12-1.34)** | **1.23** | **(1.1-1.38)** |  | **1.29** | **(1.15-1.44)** | **1.46** | **(1.28-1.66)** |  | **1.50** | **(1.33-1.68)** | **1.33** | **(1.18-1.49)** |
| Frequency of walking for at least 10 min continuously per week | never (reference) |  |  |  |  |  |  |  |  |  |  |  |  |  |  |
|  | everyday | **0.81** | **(0.72-0.9)** | 0.94 | (0.83-1.06) |  | **0.73** | **(0.67-0.8)** | **0.72** | **(0.64-0.8)** |  | **0.75** | **(0.69-0.82)** | **0.64** | **(0.57-0.72)** |
|  | 1-6 days a week | **0.84** | **(0.75-0.94)** | 0.90 | (0.78-1.03) |  | **0.85** | **(0.79-0.93)** | **0.75** | **(0.67-0.84)** |  | **0.84** | **(0.77-0.91)** | **0.70** | **(0.62-0.79)** |
| Smoking status | non-smoker (reference) |  |  |  |  |  |  |  |  |  |  |  |  |  |  |
|  | current smoker | **0.73** | **(0.64-0.82)** | 0.88 | (0.76-1.02) |  | **0.84** | **(0.77-0.91)** | 1.01 | (0.89-1.16) |  | **0.89** | **(0.82-0.97)** | 1.00 | (0.88-1.14) |

All models were adjusted for sex, age, education level, labour status, residential area, and country. CI: Confidence interval; Prevalence ratio: PR; PA: physical activity; EHIS: European Health Interview Survey; CEE: Central and Eastern Europe. Significant results are shown in bold. #Physical activity was calculated in EHIS 1 according to time spent on moderate level physical activity per week; in EHIS 2 and EHIS 3, health-enhancing physical activity (HEPA) was computed by summing up the minutes per week spent on sports, fitness or recreational (leisure) physical activities and cycling.

**
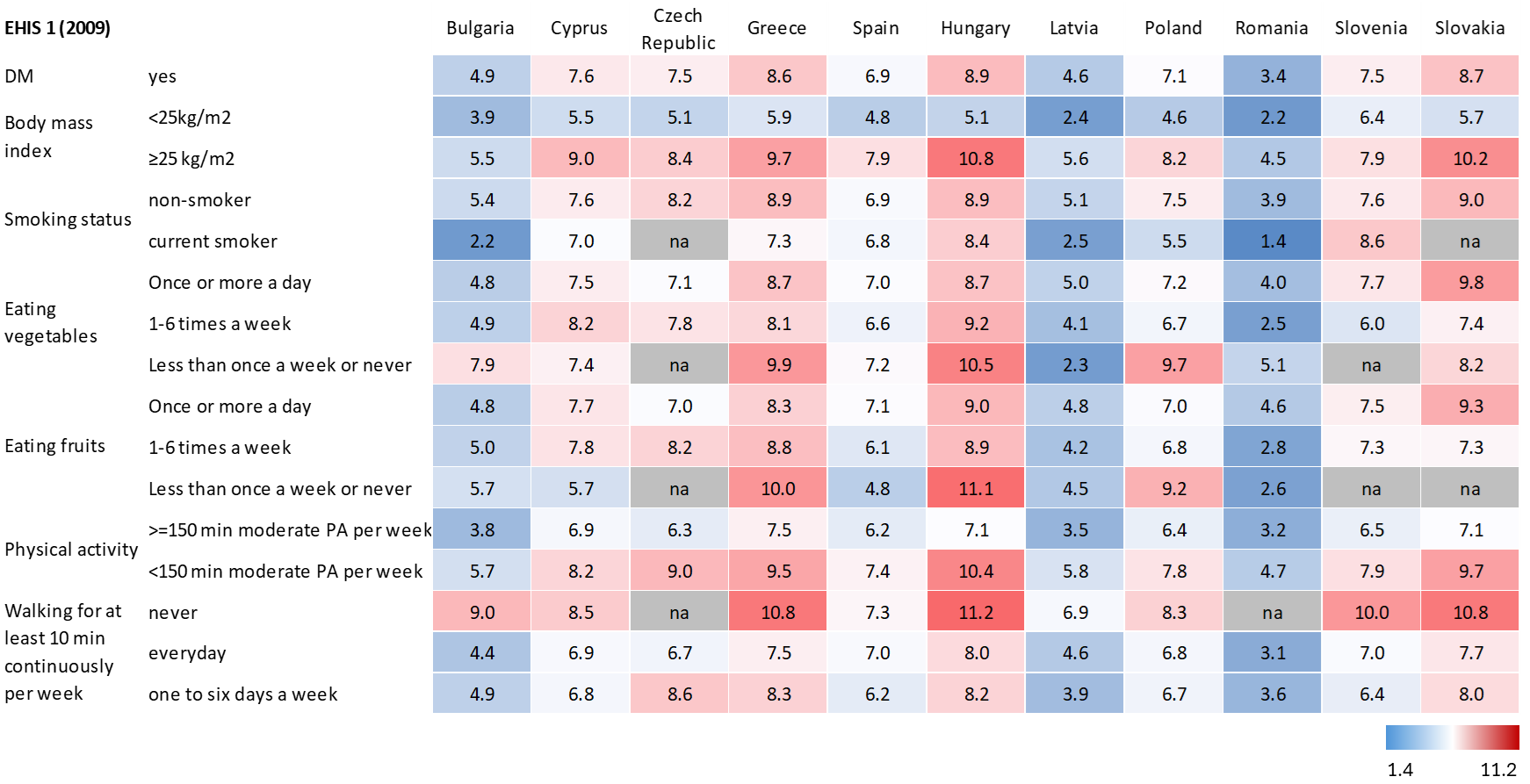
Supplementary Figures**

**Supplementary Figure S1. Age-standardised prevalence of diabetes mellitus by metabolic and lifestyle risk factors in EHIS 1.** Age-standardized prevalence of diabetes for each risk factor category was calculated for the population aged 20 years and older by 5 year age group and using the 2013 revision of the European Standard Population. “na” indicates that data were not available or that the number of respondents in at least one of the age groups was less than 5. DM: diabetes mellitus. The heatmap was created using Microsoft Excel (Microsoft 365).^1^


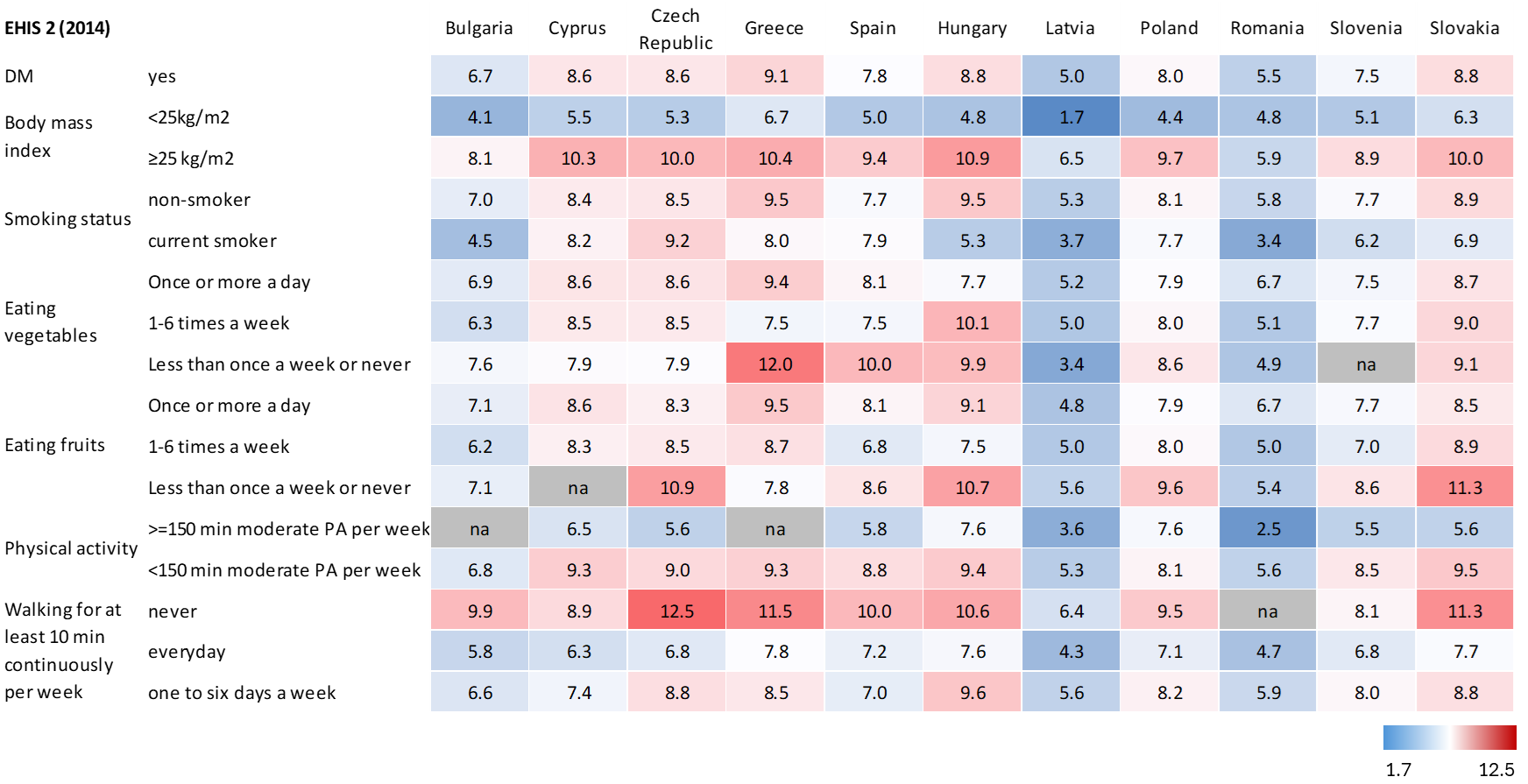


**Supplementary Figure S2. Age-standardised prevalence of diabetes mellitus by metabolic and lifestyle risk factors in EHIS 2.** Age-standardized prevalence of diabetes for each risk factor category was calculated for the population aged 20 years and older by 5 year age group and using the 2013 revision of the European Standard Population. “na” indicates that data were not available or that the number of respondents in at least one of the age groups was less than 5. DM: diabetes mellitus. The heatmap was created using Microsoft Excel (Microsoft 365).^1^


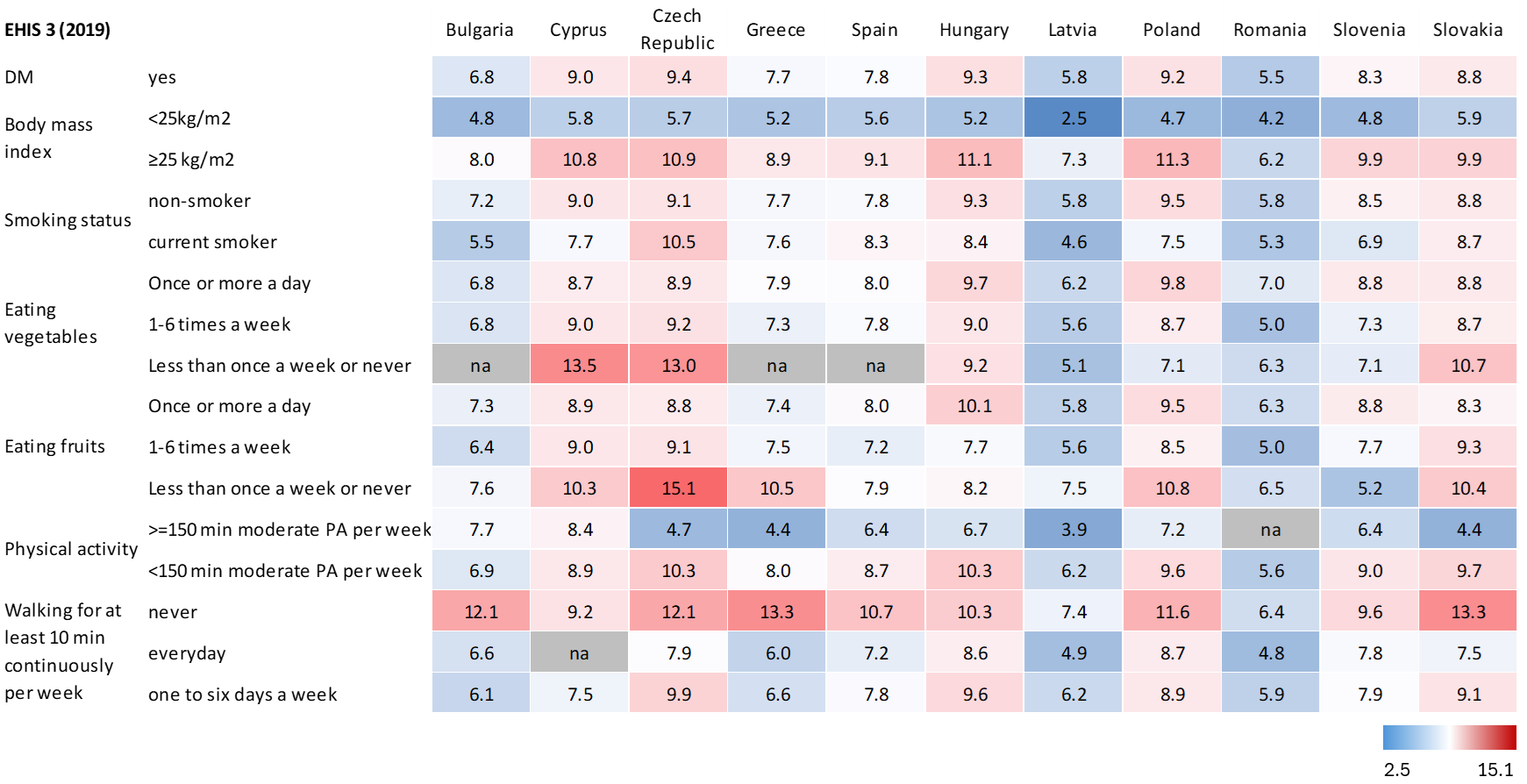


**Supplementary Figure S3. Age-standardised prevalence of diabetes mellitus by metabolic and lifestyle risk factors in EHIS 3.** Age-standardized prevalence of diabetes for each risk factor category was calculated for the population aged 20 years and older by 5 year age group and using the 2013 revision of the European Standard Population. “na” indicates that data were not available or that the number of respondents in at least one of the age groups was less than 5. DM: diabetes mellitus. The heatmap was created using Microsoft Excel (Microsoft 365).^1^

Reference

1. Microsoft Corporation. Microsoft Excel. https://office.microsoft.com/excel (2018).
